# Ezetimibe Promotes Brush Border Membrane-to-Lumen Cholesterol Efflux in the Small Intestine

**DOI:** 10.1371/journal.pone.0152207

**Published:** 2016-03-29

**Authors:** Takanari Nakano, Ikuo Inoue, Yasuhiro Takenaka, Hiraku Ono, Shigehiro Katayama, Takuya Awata, Takayuki Murakoshi

**Affiliations:** 1 Department of Biochemistry, Faculty of Medicine, Saitama Medical University, Iruma-gun, Saitama, Japan; 2 Department of Diabetes and Endocrinology, Faculty of Medicine, Saitama Medical University, Iruma-gun, Saitama, Japan; 3 Department of Diabetes, Endocrinology and Metabolism, International University of Health and Welfare Hospital, Nasushiobara-shi, Tochigi, Japan; University of Maryland, UNITED STATES

## Abstract

Ezetimibe inhibits Niemann-Pick C1-like 1 (NPC1L1), an apical membrane cholesterol transporter of enterocytes, thereby reduces intestinal cholesterol absorption. This treatment also increases extrahepatic reverse cholesterol transport via an undefined mechanism. To explore this, we employed a trans-intestinal cholesterol efflux (TICE) assay, which directly detects circulation-to-intestinal lumen ^3^H-cholesterol transit in a cannulated jejunal segment, and found an increase of TICE by 45%. To examine whether such increase in efflux occurs at the intestinal brush border membrane(BBM)-level, we performed luminal perfusion assays, similar to TICE but the jejunal wall was labelled with orally-given ^3^H-cholesterol, and determined elevated BBM-to-lumen cholesterol efflux by 3.5-fold with ezetimibe. Such increased efflux probably promotes circulation-to-lumen cholesterol transit eventually; thus increases TICE. Next, we wondered how inhibition of NPC1L1, an influx transporter, resulted in increased efflux. When we traced orally-given ^3^H-cholesterol in mice, we found that lumen-to-BBM ^3^H-cholesterol transit was rapid and less sensitive to ezetimibe treatment. Comparison of the efflux and fractional cholesterol absorption revealed an inverse correlation, indicating the efflux as an opposite-regulatory factor for cholesterol absorption efficiency and counteracting to the naturally-occurring rapid cholesterol influx to the BBM. These suggest that the ezetimibe-stimulated increased efflux is crucial in reducing cholesterol absorption. Ezetimibe-induced increase in cholesterol efflux was approximately 2.5-fold greater in mice having endogenous ATP-binding cassette G5/G8 heterodimer, the major sterol efflux transporter of enterocytes, than the knockout counterparts, suggesting that the heterodimer confers additional rapid BBM-to-lumen cholesterol efflux in response to NPC1L1 inhibition. The observed framework for intestinal cholesterol fluxes may provide ways to modulate the flux to dispose of endogenous cholesterol efficiently for therapeutic purposes.

## Introduction

Increased plasma cholesterol levels are associated with the development of atherosclerosis. Stimulation of reverse cholesterol transport (RCT), which disposes of endogenous cholesterol ultimately into the feces, can ameliorate the development. Although the hepato-biliary system has been considered as the dominant route for RCT, it has been repeatedly demonstrated that the small intestine excretes large amounts of endogenous cholesterol [1], a phenomenon designated as trans-intestinal cholesterol efflux (TICE) [2]. This pathway has been implicated as another major route of RCT in mice [1, 2]. Thus, a mechanistic understanding of TICE may provide further approaches to prevent atherosclerosis-related cardiac death.

Niemann-Pick C1-like 1 (NPC1L1), an apical transmembrane protein, plays a crucial role in cholesterol absorption [3]. Ezetimibe inhibits NPC1L1 and lowers plasma cholesterol levels [3, 4]. Furthermore, ezetimibe possesses additional favorable effect on cholesterol metabolism. The treatment of ezetimibe increased fecal neutral sterol (FNS) excretion, a surrogate marker for RCT, by 52% in humans [5]. In mice, NPC1L1 is expressed exclusively in enterocytes of the proximal small intestine [3]. Treatment of ezetimibe in mice increased FNS excretion 2.7-fold without altering hepato-biliary cholesterol disposal into the bile [6]. In the study, cholesterol originated from the diet and bile provided only less than a half of the FNS excretion. Thus, there should be the other source(s) of cholesterol in the gut that accounts for more than a half of the residual increased FNS excretion. These indicate that ezetimibe stimulates extrahepatic RCT, which would take place via the small intestine, because it is the site where the target molecule is expressed. However, it is uncertain how the inhibition of this influx transporter results in the increase.

TICE is likely a route for increased FNS excretion by ezetimibe. Direct measurements of TICE are a promising approach to elucidate its contribution for the increased FNS excretion by the drug. However, a major problem of the TICE assay is that the variability is too large to determine small changes in cholesterol flux [2]. Further careful assay settings are needed to minimize such variations. In mice, Vrins *et al*. examined the effect of ezetimibe on TICE but did not find any significant changes with large variability [7].

In the present study, we examined the effect of ezetimibe on TICE with careful normalization, and we observed that the drug increased the efficiency of TICE, explaining ezetimibe-stimulated intestinal RCT for the first time. We also attempted to elucidate how inactivation of NPC1L1 increases cholesterol efflux and its association with bidirectional cholesterol flux in the small intestine.

## Materials and Methods

### Reagents

[1, 2-^3^H]-cholesterol (53 Ci/mmol) was obtained from PerkinElmer (Waltham, MA, USA). [5, 6-^3^H]-sitostanol (50 Ci/mmol) and [22, 23-^3^H]-sitosterol (50 Ci/mmol) were obtained from American Radiolabelled Chemicals, Inc. (St. Louis, MO, USA). Other reagents were obtained from Nacalai Tesque (Kyoto, Japan) or Sigma-Aldrich (St. Louis, MO, USA) unless indicated otherwise.

### Mice

C57BL/6J and 129^+Ter^/SvJ male mice were purchased from Tokyo Laboratory Animals Science Co., Ltd. (Tokyo, Japan) and Clea Co., Ltd. (Tokyo, Japan), respectively. B6; 129S6-*Abcg5*/*Abcg8*^*tm1Hobb*^/J mice (stock number 004670), which lacked ATP-binding cassette (ABC) G5 and ABCG8 gene expression [ABCG5 and ABCG8 double knockout (DKO), The Jackson Laboratory, Bar Harbor, MA, USA], were mated in heterozygous pairs to obtain DKO mice and wild-type (WT) counterparts. Genotyping was conducted as directed by the supplier (http://jaxmice.jax.org/strain/004670.html). All the mice were examined visually a couple of times in a week, given standard chow and housed in cages in a temperature-controlled room with 12-h light cycling and used at an age of 9–25 weeks old. We euthanized mice by cervical dislocation under anesthesia when necessary. All animal experiments were approved by the Animal Care Committee of Saitama Medical University.

### Trans-intestinal cholesterol efflux assay

TICE assays were performed in accordance with van der Velde *et al*. [2] with some modifications. We administered C57BL/6J mice 100 μl Intralipid (20% emulsion) with or without 50 μg ezetimibe the day before the assay by gavage, because ezetimibe undergoes glucuronidation in the intestine and liver, and both the parent compound and its glucuronide localize to the brush border membrane (BBM) of the small intestine with the pharmacological activity [8]. The same reagent was given again 3 h before the assay. We anesthetized C57BL/6J mice with an intraperitoneal injection of a cocktail of ketamine (65.2 mg/kg) and xylazine (18.5 mg/kg), cannulated the right jugular vein with MicroRenathane Implantation Tubing (MRE-025, Braintree Scientific Inc., Braintree, MA, USA), and ligated the common bile duct with a 7–0 surgical suture.

We then ablated and incised the upper jejunum to cannulate at 5 cm distal from the cardia with a silicone tube (external diameter, 2.5-mm). We sealed off the gap between the luminal wall and the outside of silicone tube using surgical glue to prevent the luminal contents in the stomach and duodenum from leaking into the cannulated segments. Subsequently, we ablated and incised again the jejunum at 4–7 cm distal from the upper cannulating locus, inserted a silicone tube of 3-mm external diameter, and sealed off the gap as well. The resulting cannulated segment was separated from the luminal flow of the other intestinal tracts but not from the circulation. Ablation was done to prevent bleeding from the incisions.

One hundred μl Intralipid (20% emulsion) containing 10 μCi ^3^H-cholesterol tracers and 50 μg of ezetimibe or vehicle (ethanol) was injected into the jugular vein, and the cannulated intestinal segment was perfused with Krebs-HEPES buffer containing 10 mmol/l taurocholic acid and 2 mmol/l phosphatidylcholine (a perfusion buffer) at a flow rate of 0.2 ml/min for 1 h. The perfused aliquots were collected for 10 min each. After the perfusion, blood was collected, and the remaining blood in the circulation was flushed out with saline. The small intestinal segment perfused was excised, and the tissue was solubilized in a tissue solubilizer (SOLVAVLE, PerkinElmer). ^3^H-decay per minute (DPM) of radioactivity in the samples was mixed with Hionic-Fluor and Ultima Gold scintillation cocktails (PerkinElmer) for solubilized tissues and the other samples, respectively, and counted using a scintillation counter (Tri-Carb 2910TR, PerkinElmer). We estimated TICE as follows:

Tracer count in the perfusates (DPM) / length of the small intestine perfused (cm) / tracer count of serum samples (DPM).

For TICE assay, we gave mice 50 μg ezetimibe twice orally. In addition, we gave the same dose via the jugular vein injection just before the perfusion, because we initially thought that ezetimibe could stimulate TICE by activating possible basolateral machineries of the epithelial cells in the small intestine. The overall dosage is estimated 6 mg/kg; mouse body weight as 25 g. For medical treatment, 140 μg/kg per day of ezetimibe is usually prescribed (assumed as 70 kg human body weight). Because the murine NPC1L1 has more than 1,000-fold lower binding affinity to ezetimibe than simian NPC1L1 (The ED_50_ value for humans was not available.) [9], the treatment we used was unlikely a supra-pharmacological dosage.

### Monitoring circulating ^3^H-cholesterol levels infused via the jugular vein

We infused ^3^H-cholesterol tracer together with 100 μl of Intralipid into C57BL/6J mice via the jugular vein and ligated the bile duct as described above, followed by blood sampling (5 μl each) from the tail vein at 10-min intervals up to 60 min. The blood samples were diluted in 100 μl of saline, bleached with H_2_O_2_, and counted for ^3^H radioactivity.

### Luminal perfusion assay of the upper jejunum

We developed a modified TICE assay, designated as luminal perfusion assay, to detect BBM-to-lumen cholesterol efflux as follows: We gave mice 0.1 ml triolein, instead of Intralipid for TICE assay, to mimic physiological lipid ingestion, twice as described above (day -1 and day 0) with or without ezetimibe (1, 5, or 50 μg). Three hours after the second oral infusion with 5 μCi of ^3^H-cholesterol tracer, we opened the abdomen of anesthetized mice and cannulated the upper jejunum as performed in TICE assay. The lumen of the cannulated segment was washed with 3 ml of Krebs-HEPES buffer from the proximal to the distal end at a flow rate of 0.25 ml/min for 12 min. Washing solution aliquots were collected for 4 min each (1 ml/tube). Then, the buffer in the lumen was replaced with the perfusion buffer for 2 min, followed by perfusion with the same buffer at a flow rate of 0.05 ml/min for 1 h. All the solutions used for the luminal perfusion assays were pre-equilibrated to room temperature (20–25°C). Perfusate aliquots were collected for 15 min each, and designated as fractions #1, #2, #3, and #4. Each fraction was counted for ^3^H radioactivity. After the experiment, blood was collected, and the remaining blood in the circulation was flushed out with saline. The perfused intestinal segment was excised, solubilized in SOLVAVLE, and counted for ^3^H radioactivity. We determined DPM per cm for each intestinal segment.

For luminal perfusion assays with ^3^H-sitosterol or ^3^H-sitostanol tracers, we added 0.5 mg of a plant sterol cocktail (Thermo Fisher Scientific Inc., Waltham, MA, USA, cat.no.13272, Lot. A0265923; β-sitosterol, 78.2%; β-sitostanol, 10.6%; campesterol, 7.5%; campestanol, 0.9%) to the 5 μCi tracer-containing second triolein infusate as a sterol carrier, because there is virtually no plant sterol in the lumen. We started the efflux phase 2 h and 1 h after the second triolein infusion for sitosterol and sitostanol tracers, respectively. At the time points, peak tracer labelings for sitosterol and sitostanol tracers, respectively, were detected in the jejunum (data not shown), consistent with a previous report [10].

### *Ex vivo* luminal perfusion assay

To estimate non-specific liberation of cholesterol from the intestinal epithelia, we mimicked a luminal perfusion assay *ex vivo*. Three hours after the ^3^H-cholesterol infusion, we excised 5-cm upper jejunal segments that corresponded to the region used in the luminal perfusion assay, immersed the segments in cold Krebs-HEPES buffer, cannulated the proximal side using a silicone tube, and washed the lumen with 3 ml of Krebs-HEPES buffer. Then, the segments were opened longitudinally and immersed in 3 ml of a perfusion buffer, which was the same buffer used in the efflux phase of the luminal perfusion assay, for 1 h on ice. We counted ^3^H tracer radioactivity of liberated into the buffer and retained in the tissue. The *ex vivo* luminal cholesterol efflux (%) was calculated as follows:

Tracer counts in the perfusion buffer (DPM) / tracer counts retained in the intestinal segment (DPM) ×100.

### Alkaline phosphatase activity assay

Alkaline phosphatase activity was measured as a marker of epithelial cell damage [11]. Briefly, 10 μl of each luminal perfusates (fractions #1–#4) were mixed with 190 μl of a substrate buffer and incubated for 5 min at 37°C. We calculated the mean absorbance at 405 nm of the four fractions and showed the absorbance as alkaline phosphatase activity in luminal perfusates.

### Efflux of cholesterol in murine primary enterocytes

Murine primary enterocytes were isolated from jejunal segments in accordance with the method reported by Haidari *et al*. [12]. We infused ^3^H-cholesterol-containing triolein into C57BL/6J mice by gavage as described above and allowed to absorb the tracer for 3 h. Then, the jejunal segments were excised and cut into 5-mm lengths, placed in ice-cold Matrisperse (BD Biosciences, Franklin Lakes, NJ, USA), and incubated on ice for 4 h. Enterocytes were liberated by agitating mildly, passed through 100-μm nylon mesh, and washed twice in ice-cold PBS. The cells were incubated in Dulbecco’s modified Eagle’s medium (DMEM) containing 20% heat-inactivated fetal calf serum (FCS) for up to 2 h. The culture media and cells were recovered at the indicated time points, and ^3^H radioactivity was counted. We calculated the percentage of cholesterol radioactive tracer in the culture media against the sum of tracer counts in the media and the cell lysates, which was expressed as “cellular efflux (%)”. The purity of the enterocytes obtained was examined by staining for alkaline phosphatase activity using ELF-97 (Life Technologies, Carlsbad, CA, USA), a phosphatase substrate that emits fluorescence upon dephosphorylation. Cells were observed using a fluorescence microscope (Zeiss Axioplan 2 imaging; MOT, Carl Zeiss, Jena, Germany) and images were captured using AxioVision software (Carl Zeiss).

### Distribution of orally-given cholesterol tracer in C57BL/6J mice

Ezetimibe and ^3^H-cholesterol were given orally to mice as described for the luminal perfusion assay. The abdomen was opened under anesthesia, blood was obtained from the inferior vena cava, and then the remaining blood in the circulation was flushed out using cold saline. The stomach was excised, and the luminal contents were rinsed in 10 ml of cold saline for tracer counting. The small intestine was cut equally into three pieces (designated as proximal, medial, and distal segments). The segments were opened longitudinally, and the small intestinal luminal contents were obtained by rinsing each segment in 10 ml of cold saline. The luminal contents of the cecum were also obtained as well. The remaining intestinal tissues were lyzed using SOLVAVLE. Whole liver samples were weighed, the portions (0.1–0.15 g) were lyzed, and counted for ^3^H-radioactivity to estimate total count in the liver. Blood was clotted to obtain serum samples. Serum volume in individual mice was estimated [body weight (g) × 0.08 (a factor for blood volume vs. body weight) × (1–0.6) (0.6 was used as murine hematocrit)] and used to adjust the DPM for total serum. The total DPM in the liver and serum was used to estimate total transit of ^3^H-cholesterol into the body.

### Radioactivity measurement in isolated enterocytes

C57BL/6J mice were given an intragastric load of 0.1 ml triolein and fasted overnight. Then 0.1 ml triolein containing 5 μCi ^3^H-cholesterol was given again and allowed to absorb for 3 h. Ezetimibe (1, 5, or 50 μg) or vehicle (ethanol 2.5 μl) was added to each triolein infusate. The enterocytes were obtained, lyzed in a lysis buffer, and assayed for protein concentration as described above. ^3^H radioactivity in enterocytes was counted as described above. Specific radioactivity (DPM/mg protein) in the lysates was calculated.

### HepG2 cells and transfections

The coding regions of NPC1L1 was obtained from cDNAs from Caco-2 cells (ATCC no. HTB-37, American Type Culture Collection, Manassas, VA, USA) and subcloned into a pcDNA3.1 expression vector. One day after seeding HepG2 cells (stock no. JCRB1054, JCRB Cell Bank, Tsukuba, Japan) into 24-well plates, the vector was transfected into the cells using Lipofectamine 2000 (Life Technologies) in accordance with the manufacturer’s instructions.

### Medium-to-cell cholesterol transit in HepG2 cells

One day after transfection, we discarded the culture medium, added 0.5 ml DMEM containing 1 μCi ^3^H-cholesterol, 0.1 mmol/l phosphatidylcholine, 0.05 mmol/l cholesterol, and 0.5 mmol/l taurocholic acid, and incubated for 2 h. Medium-to-cell ^3^H-cholesterol transit (%) was calculated as below.

Tracer count in cell lysate (DPM) / total cholesterol count given to medium (DPM) × 100.

Total cholesterol (both non-esterified and esterified) concentration was determined using a Cholesterol/Cholesteryl Ester Quantitation Kit (Biovision Inc., San Francisco, CA, USA). Protein concentration was determined using a BCA Protein Assay kit (Thermo Fisher Scientific, Inc.).

### Western blotting

#### Sample preparations

HepG2 cells were scraped off from the dishes after the cells were washed twice with cold PBS. Total cellular protein was precipitated using 10% trichloroacetic acid. The precipitate was dissolved and denatured with 80 μl of a cocktail of 9 mol/l urea, 2% Triton X-100, and 1% dithiothreitol. Then, 20 μl of 10% lithium dodecyl sulfate was added and neutralized with Tris. Cytosolic and membranous protein was separated using a Mem-PER Eukaryotic Membrane Protein Extraction Kit (Thermo Fisher Scientific Inc.).

#### Western blotting

Proteins obtained were separated on 8% polyacrylamide gels, and then stained with QuickBlue Staining Solution (Biodynamics Laboratory Inc., Tokyo, Japan) or transferred to polyvinylidene difluoride membranes (GE Healthcare, Buckinghamshire, UK). Anti-NPC1L1 polyclonal antibody (1 μg/ml, cat.no. HPA018105, Sigma-Aldrich), anti-ABCG5 monoclonal antibody (1:500, clone no. EPR6203 from rabbit; cat.no. GTX63251, GeneTex Inc. Irvine, CA, USA), and anti-ABCG8 polyclonal antibody (2 μg/ml, cat.no. GTX30466, GeneTex) were used as primary antibodies. Horseradish peroxidase-conjugated anti-rabbit IgG antibody (1:1,000, GE Healthcare) was used as the secondary antibody. Bands were visualized using a chemiluminescence kit (ECL, Western Blotting Detection Reagents; GE Healthcare) with high-performance chemiluminescence film (Hyperfilm ECL, GE Healthcare) or ChemiDoc MP system (Bio-Rad laboratories, Hercules, CA, USA). MagicMark, a chemiluminescent protein size marker, was used. We adjusted the signal levels when necessary with software (Photoshop, Adobe Systems Incorporated, Park Avenue, San Jose, CA, USA) to enhance visibility of bands.

### Differentiation of Caco-2 cells on filter membranes

Caco-2 cells were differentiated on filter membranes (0.4 μm Individual Cell Culture Inserts, BD Falcon, BD Biosciences, San Jose, CA, USA) [13]. Briefly, Caco-2 cells were plated at a density of 5 × 10^4^ cells per cm^2^ and grown in a humidified 95% atmosphere / 5% CO_2_, at 37°C in DMEM containing 25 mmol/l glucose and GlutaMAX (Product No. 10564–011, Invitrogen) and 20% FCS. Seven days after the culture, the cells were cultured in asymmetric conditions, with DMEM containing 5 mmol/l glucose and GlutaMAX in the upper compartment (apical medium) and DMEM containing 20% FCS in the lower compartment (basolateral medium) for 1 week. Then, the basolateral media was replaced with DMEM containing ITS supplement (Life Technologies Inc., Carlsbad, CA, USA). Penicillin/streptomycin (100 IU/ml and 100 μg/ml, respectively) and 1% non-essential amino acid solution (100×, Thermo Fisher Scientific Inc.) were added to all the above media.

### Microscopic analysis

Fluorescence immunohistochemistry was performed as described previously [14, 15]. In brief, differentiated Caco-2 cells were fixed with 1% paraformaldehyde for 1 h at room temperature and used for immunostaining. Anti-human NPC1L1 antibody (see “Western blotting”) and Alexa Fluor 488 goat anti-rabbit IgG antibody (1:500, Life Technologies) were used as the first and second antibodies, respectively. 7-amino-actinomycin D was used for counterstaining of nuclei. Fluorescence images were obtained using a confocal microscope (Radiance 2000MP, Bio-Rad laboratories). Electron microscopy of differentiated Caco-2 cells was conducted as described previously [14].

### Preparation of lipid micelles

Lipid micelles were prepared as described previously [13, 16]. In brief, stock solutions of oleic acid, 2-mono-oleylglycerol, lysophosphatidylcholine, and non-esterified cholesterol were mixed in a sterile glass tube and dried under a stream of nitrogen and stored at –80°C. The resulting dried lipids were dissolved in 83 μl of a sterile solution of 24 mmol/l taurocholic acid (Nacalai Tesque, Kyoto, Japan) in serum-free DMEM (5 mmol/l glucose and GlutaMAX, Invitrogen, CA, USA) and the tube was placed in an ultrasound bath (UT-104, Sharp Co., Osaka, Japan) for 10 min at room temperature. Then 1 ml of serum-free medium was added to the lipid micelles, and brief ultrasound treatment was repeated. The final lipid concentrations were 0.6 mmol/l oleic acid, 0.05 mmol/l cholesterol, 0.2 mmol/l 2-mono-oleylglycerol, 0.2 mmol/l lysophosphatidylcholine and 2 mmol/l taurocholic acid, which mimics the composition of post-digestive duodenal micelles [17]. Lipid micelles were used within 2 h after preparation.

### Medium-to-cell cholesterol transit in differentiated Caco-2 cells

We prepared DMEM containing lipid micelles, and 1 μCi/ml ^3^H-cholesterol and added to differentiated Caco-2 cells from the apical side. The cell were incubated at indicated periods, scraped off from the inserts, and counted for ^3^H tracer activity in the cells. Percent of medium-to-cell cholesterol transit was calculated as above.

### Quantitative RT-PCR

Total RNA samples were isolated from the murine upper jejunal segments and differentiated Caco-2 cells. Total RNA from human duodenum was obtained from Agilent Technologies Inc. (MVP total RNA human duodenum, cat.no. 540131; Santa Clara, CA, USA). They were reverse-transcribed using a QuantiTect Rev. Transcription Kit (QIAGEN, Valencia, CA, USA). Quantitative expression analysis was performed with an ABI PRISM 7900 Sequence Detector (Life Technologies) using SYBR green technology. The cycle threshold (Ct), corresponding to the number of cycles after which the target-DNA concentration increase becomes exponential, was monitored. Results were analyzed using SDS 2.1 Software (Applied Biosystems). We used *B2M* (β2 microglobulin) and *18s* ribosomal RNA as housekeeping genes for specimens from human and mice, respectively. All reactions were done in duplicate for each sample. The primer sets used are listed in S1 and S2 Tables.

### Statistical analysis

Data are shown as plots with indications of means and medians when the data show parametric and non-parametric distributions, respectively. Data are also shown as means ± standard error of the mean (SEM) unless indicated otherwise. Means were compared using the Student’s *t*-test, or the Mann—Whitney *U* test if the data distribution was non-parametric. For multiple comparisons, we used Dunnett’s test or Tukey's Honestly Significant Difference test. *p* < 0.05 was considered to be significant. Statistical analyses were performed using JMP ver.7.0 (SAS Institute, Inc., Cary, NC, USA).

## Results

### Ezetimibe increases trans-intestinal cholesterol efflux efficiency

**Fig 1:**
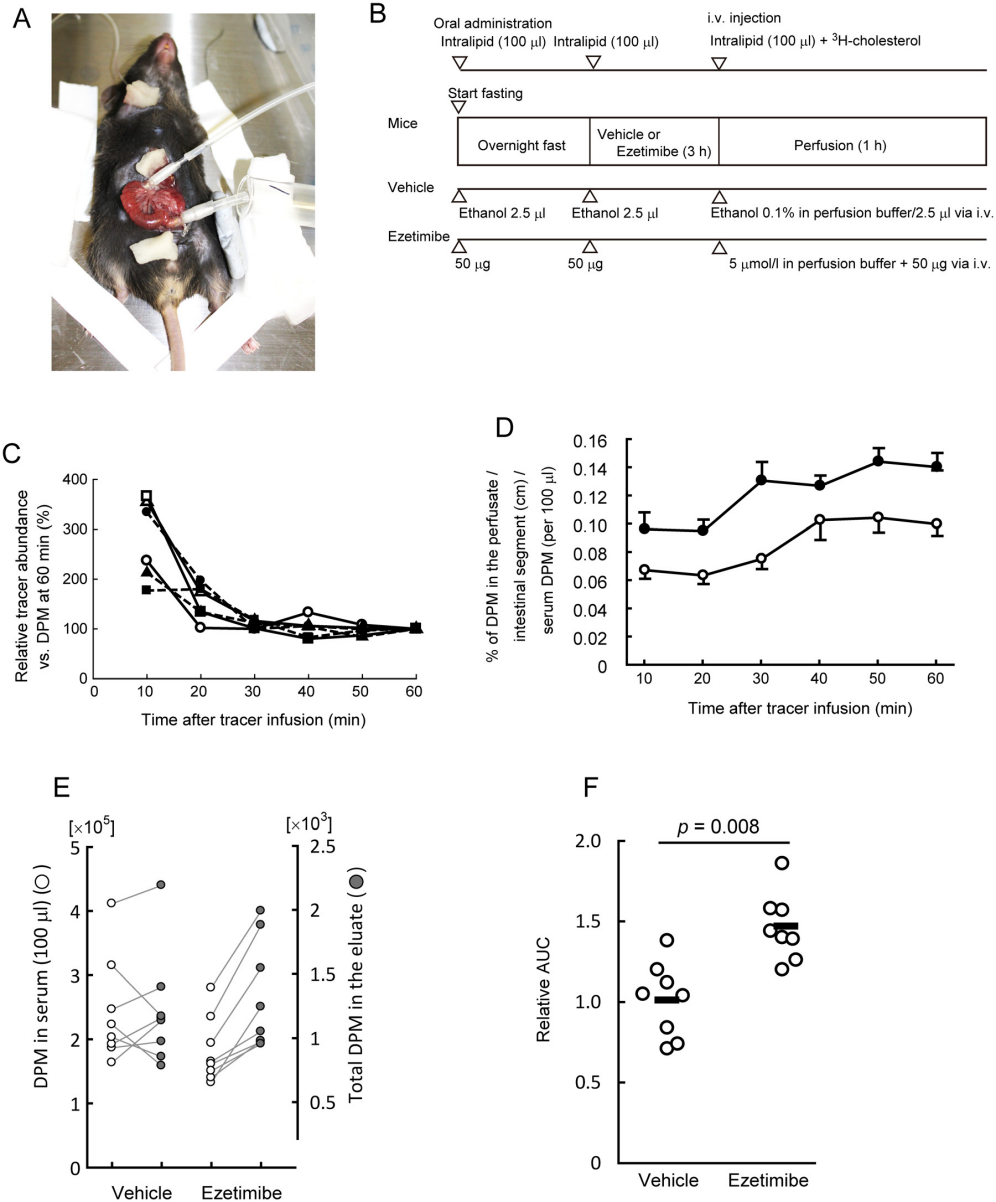
Inhibition of NPC1L1 increases trans-intestinal cholesterol efflux (TICE). *A*, An example of the TICE assay settings. *B*, An illustrated protocol for TICE assay. Assay time course is indicated from left to right. Arrow heads show the time points when reagents were given to mice. *C*, Decay per minute (DPM) counts in 5 μl blood obtained at the indicated time points after ^3^H-cholesterol intravenous infusion via the jugular vein. The DPM counts for individual mice were normalized against that at 60 min. Each symbol indicates an individual assay result. Infused tracer seemed to be equivalent in the circulation approximately 30 min after the infusion. *Open symbols*, vehicle controls; *closed symbols*, mice treated with ezetimibe. *D*, Ezetimibe treatment increased TICE. *Open circles*, vehicle; *closed circles*, 50 μg ezetimibe treated. Plots and error bars show mean and SEM (n = 8), respectively. *E*, Plots for ^3^H-DPM counts in sera (*left*, *open circles*) and those in total perfusates (*right*, *gray circles*). *F*, Comparison of the area under the concentration-time curve (AUC) of *D*. Each plot shows an individual assay result. *P* value was obtained using the Student’s *t*-test.

In the quantification of TICE (Fig 1A and 1B), attempts were made to minimize assay-to-assay variation at first. The circulating tracer abundance would affect the resulting DPM in the perfusate, although it varied between mice even when the similar tracer amount was injected. Monitoring the circulating tracer abundance for 1 h showed that the level was steady within 30 min after the intravenous infusion, and thereafter it was sustained until the end of the assay period (Fig 1C). We thought that tracer count in serum obtained at the end of TICE assays could be used as a surrogate quantity for circulating tracer abundance over the period (the denominator for TICE calculation; see the vertical axis of Fig 1D). We also normalized the value to the length of segments (cm), because DPM level in the perfusate would be proportional to the cannulated intestinal length. The lengths did not differ considerably among the groups (data not shown), excluding assay bias with the lengths.

Monitoring the normalized value of TICE showed that the efflux rate was increased with ezetimibe (Fig 1D). The DPM values in serum showed that ezetimibe did not alter the circulating level of DPM significantly (Fig 1E). The area under the concentration-time curve (AUC) analysis of data shown in Fig 1D confirmed a 45% increase of TICE by administering ezetimibe (Fig 1F).

When we measured DPM in the intestinal segments perfused after TICE assays, approximately 0.2% to 0.3% of total tracer infused was detected (0.27 ± 0.04% for vehicles and 0.22 ± 0.03% for ezetimibe-treated; mean **±** SEM), showing that there was substantial transit of cholesterol tracer from the circulation to the small intestine irrespective in the presence or absence of ezetimibe.

### Inhibition of NPC1L1 increases brush border membrane-to-lumen cholesterol efflux

**Fig 2:**
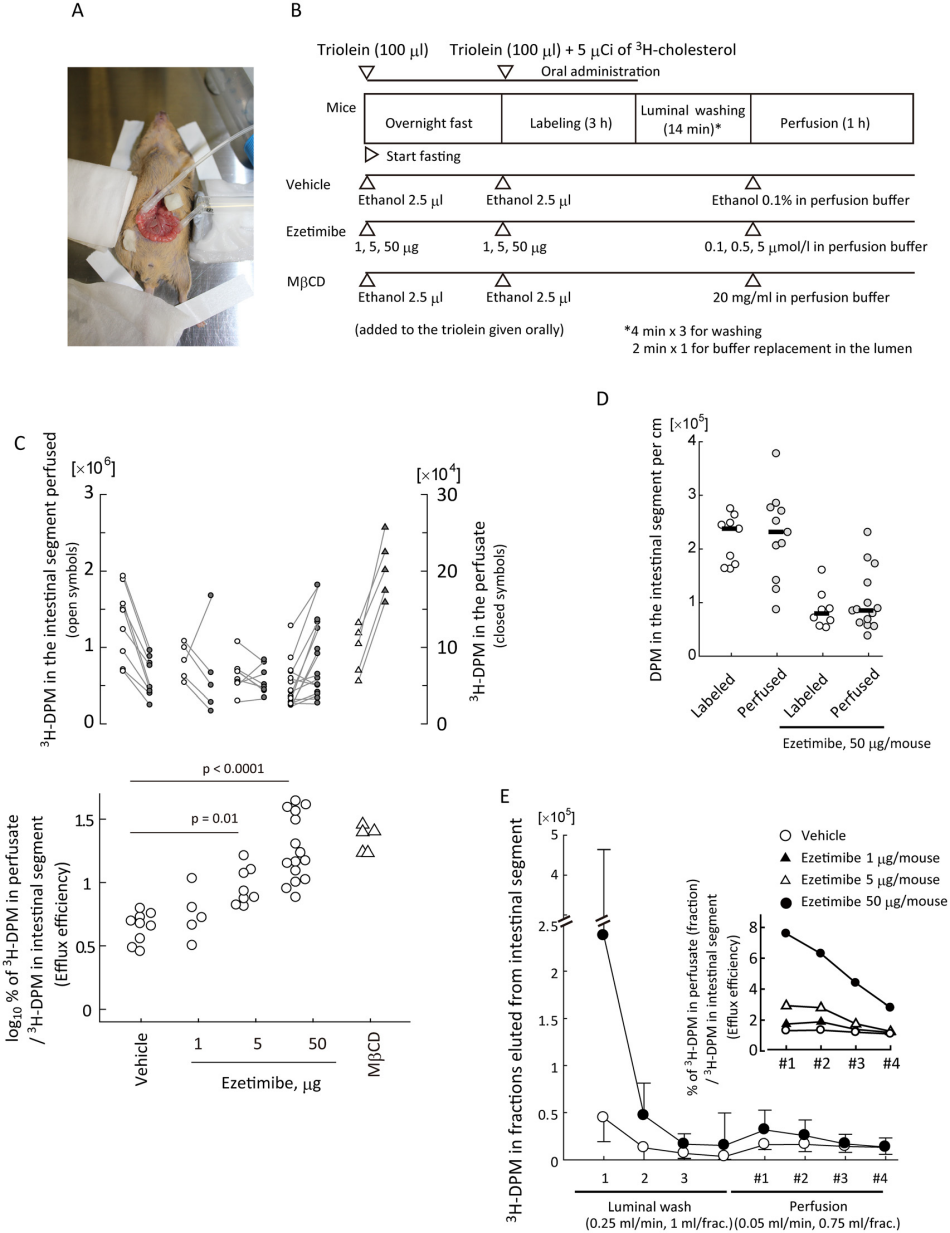
Ezetimibe increases brush border membrane-to-lumen cholesterol efflux. *A*, An example of jejunal cannulation for the luminal perfusion assay (129^*+Ter*^/SvJ mouse). *B*, An illustrated protocol for the luminal perfusion assay. Assay time course is indicated from left to right. Arrow heads show time points when reagents were given to mice. *C*, *Upper*; Plots for ^3^H-decay per minute (DPM) counts in the perfusate (*right*, *gray circles*) and the perfused intestinal segment (*left*, *open circles*). *Lower*; ^3^H-DPM in the perfusate was divided by that in the intestinal segment perfused to obtain a ratio (%), which was then converted to a common logarithm, and compared using Dunnett’s test. *Circles* show vehicle controls or ezetimibe treatment (1–50 μg). *Triangles*, methyl-β-cyclodextrin (MβCD), a cholesterol absorbent; 20 mg/ml in the perfusion buffer. Each plot shows an individual assay result. *D*, Intestinal ^3^H-DPM abundance did not change apparently during luminal perfusion assay. Intestinal segments were obtained 3 h after the labeling with ^3^H-cholesterol (*open circles*) or after luminal perfusion assay (*gray circles*) for DPM count (separate experiments from Fig 2C). Bars show median DPM of each group. Each plot shows an individual assay result. The data for the perfused columns were obtained from the data shown in *C*, which were divided with the respective intestinal lengths perfused (cm). *E*, Elution pattern of ^3^H-cholesterol from the intestinal lumen with 0–50 μg/mouse ezetimibe in luminal perfusion assays of *C*. The *inset* shows fractional perfusate/intestine ^3^H-DPM ratios (fractional efflux efficiency).

Because NPC1L1 is a brush border protein, increased efflux in TICE assays suggested a possibility that ezetimibe increases BBM-to-lumen cholesterol efflux. To assess this hypothesis, we performed luminal perfusion assays (Fig 2A and 2B) in the murine jejunum in the presence or absence of ezetimibe, in which ^3^H-cholesterol was given orally to label the intestinal epithelial BBM with tracers from the luminal side. This should label the BBM with cholesterol tracer even in the presence of ezetimibe, because cholesterol can enter the membranous components independent of NPC1L1 [18]. We evaluated changes in the ratio of ^3^H-DPM in the perfusate to ^3^H-DPM in the intestinal segment perfused, and found that ezetimibe increased the ratio in a dose-dependent manner (Fig 2C), reaching 3.5-fold with a 50 μg dosage.

### Alternation of ^3^H-cholesterol abundance in intestinal segments during the luminal perfusion assay

Ideally, tracer abundance in intestinal segments and that appeared in the lumen are to be compared sequentially over the assay period in luminal perfusion assay. However, DPM in intestinal segments could not be measured non-invasively; thus, we used the DPM from the segment obtained at the end of the assay as the denominator to estimate the efflux. A concern was the reliability, because it was uncertain how much change occurred in DPM abundance of the small intestinal segments during the perfusion period. We determined radioactivity in small intestinal segments before and after perfusion, and found that there was no apparent change in the median values (Fig 2D). This indicates that DPM value in the small intestine was stable at least during the assay; thus, the value obtained at the end of the assay can be reliable to estimate the tracer abundance over the assay period.

Because cholesterol transit from enterocytes to the circulation was slow enough to exclude the DPM loss in the intestinal segment during the assay (1 h) (Fig 2D and Ref. [19]), the ratio of DPM in the perfusate to DPM in the intestinal segment indicates what amount of cholesterol tracer in the intestinal segment was effluxed during the period. With these assumptions, we refer the value of the ratio of ^3^H-DPM in the perfusate to ^3^H-DPM in the intestinal segment perfused (%) as “efflux efficiency” hereafter.

### Increased cholesterol efflux originates from enterocytes

Fig 2E shows the DPM values of each fraction in the luminal perfusion assays. The mice treated with ezetimibe had greater ^3^H-cholesterol abundance in the lumen than vehicle controls at the beginning, which might be a possible assay bias. We thought that increased BBM-to-lumen efflux resulted in the accumulation of ^3^H-cholesterol tracer in the lumen in the ezetimibe-treated mice. Furthermore, we did not think that the greater retention of the tracer in the lumen and the mucus layer of the small intestine falsely resulted in the greater efflux efficiencies with ezetimibe (Fig 2C), because of the following observations.

#### Residual ^3^H-cholesterol tracer in the lumen was efficiently removed by pre-washing

Washing the cannulated intestinal segment with 3 ml buffer efficiently eliminated remaining luminal tracer before the subsequent perfusion step (Fig 2E); thus, such tracer appeared in the perfusate was unlikely to originate from a liberation of the trapped ^3^H-cholesterol in the lumen.

#### ^3^H-cholesterol in the perfusate was not originated from those retained in the mucus layers of the small intestinal segments

**Fig 3:**
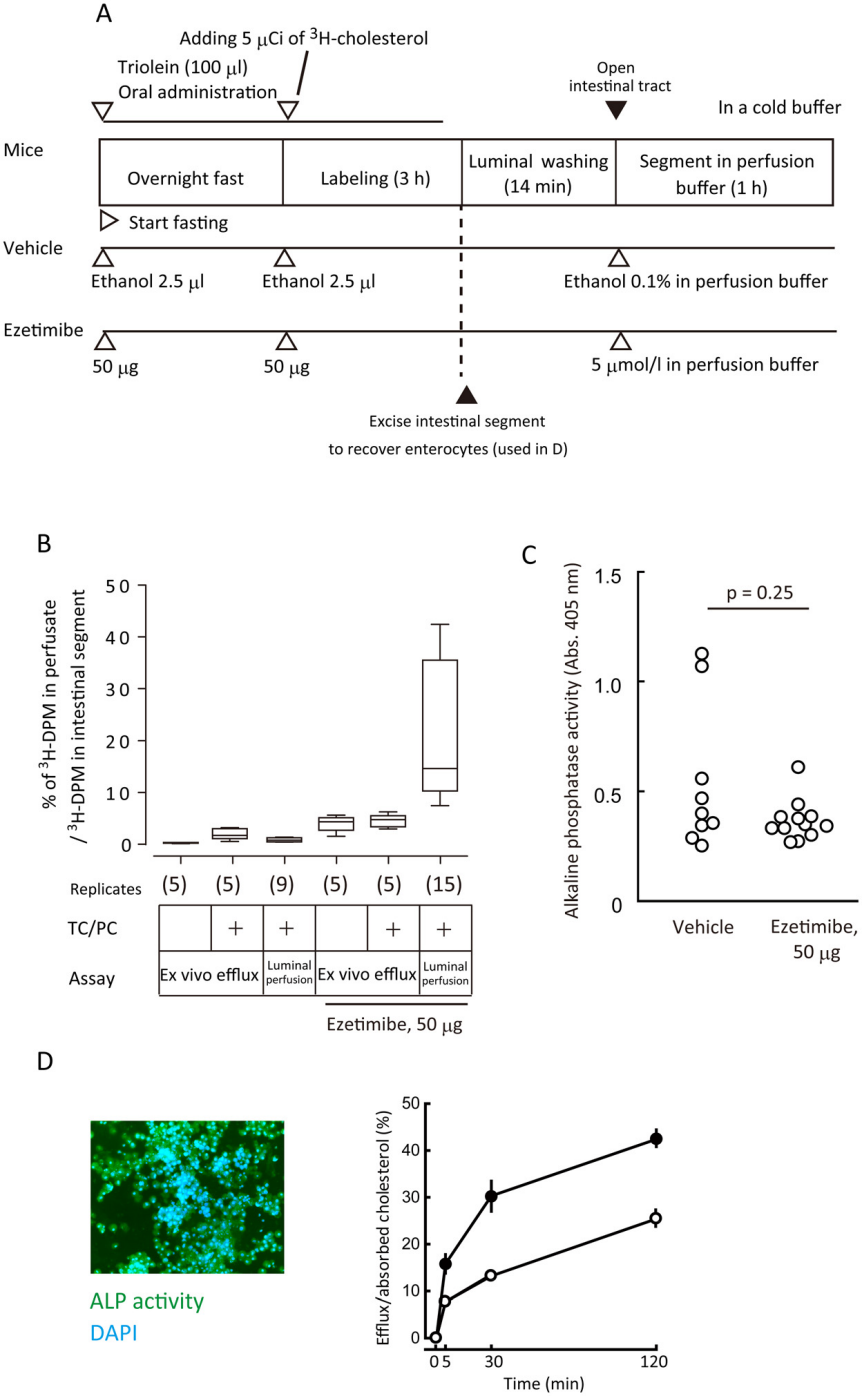
Intestinal cholesterol efflux is a biologically active phenomenon. *A*, An illustrated protocol for the *ex vivo* cholesterol efflux assay and for the recovery of enterocytes. Assay time course is indicated from left to right. Open arrow heads show time points when reagents were given to mice. Closed arrow heads indicate events for processing of the small intestine. *B*, Comparison of luminal perfusion assay and *ex vivo* cholesterol efflux assay on the elution of cholesterol from intestinal segments. TC/PC indicates taurocholic acid and phosphatidylcholine. Bars indicate the mean percentages of ^3^H-decay per minute (DPM) counts of perfusate/intestinal segment in the luminal perfusion assay and ^3^H-DPM of elution/intestinal segment in *ex vivo* cholesterol efflux assay. *Box lines*, median (*center*), 25th percentile (*lower*), and 75th percentile (*upper*); *error bars*, 10th and 90th percentiles. Numbers in the parentheses show assay replicates. *C*, There was no significant difference in alkaline phosphatase (ALP) activity in the perfusates with or without ezetimibe treatment. Data were compared using the Mann—Whitney *U*-test. Each plot shows the result from an individual luminal perfusion assay. *D*, *Left*, green indicates ALP activity, an enterocyte-specific marker in the small intestine, using a fluorogenic ALP substrate, ELF-97. *Right*, Kinetics of cholesterol efflux in murine primary enterocytes. Enterocytes from mice treated with (*closed circles*) or without (*open circles*) ezetimibe (50 μmol/l) were incubated for up to 2 h. Data are shown as mean ± SEM of triplicate assays.

Another possibility is that the tracer retained in the lumen and/or mucus layer supplied ^3^H-cholesterol liberation to increase the efflux falsely. To exclude this, we performed a luminal perfusion assay *ex vivo* on ice (Fig 3A). At this temperature all cellular membrane trafficking is completely blocked, but it would not limit cholesterol liberation from the lumen and the mucus layer. As a result, we did not observe accountable increase in ^3^H-cholesterol liberation in the efflux assay *ex vivo* (Fig 3B).

#### Ezetimibe does not affect intestinal cell sloughing

Ezetimibe treatment did not affect alkaline phosphatase activity in the perfusate, confirming that the increased DPM count in the perfusate by ezetimibe did not originate from cell debris due to a toxic effect on cells lining the intestinal lumen (Fig 3D).

#### Ezetimibe increased cholesterol efflux in isolated murine enterocytes

*In vitro* examination of cholesterol efflux efficiency with purified enterocytes showed that ezetimibe doubled efflux in ^3^H-cholesterol-labelled enterocytes (Fig 3C). Collectively, these observations indicate that the increased efflux was an enterocyte-mediated phenomenon.

### Lumen-to-brush border membrane transit is faster than enterocyte-to-circulation transit in cholesterol absorption

Intestinal cholesterol absorption means net transit of cholesterol from the lumen to the circulation. The transit can be divided into ‘uptake’ and ‘export’ steps. ‘Uptake’ refers the transfer of cholesterol from luminal micelles to the BBM (lumen-to-BBM). ‘Export’ indicates to the transfer of cholesterol from the enterocytes to the circulation (enterocyte-to-circulation).

**Fig 4:**
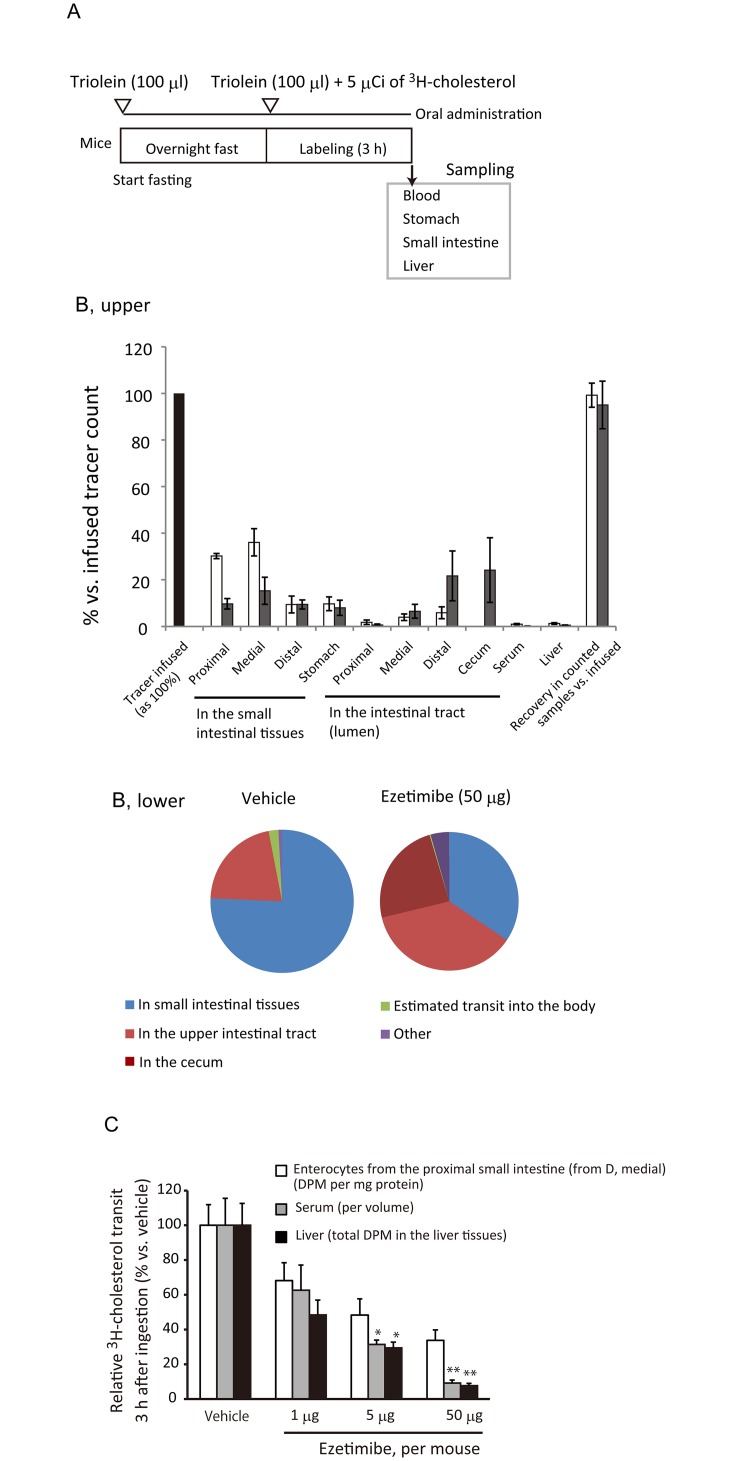
Effect of ezetimibe on cholesterol transit in mice. *A*, An illustrated protocol for the ^3^H-cholesterol distribution assay. *B*, *Upper*; ^3^H-decay per minute (DPM) count distribution 3 h after ^3^H-cholesterol was given orally to C57BL/6J mice. Bars indicate mean and the standard deviation (*n* = 5 for vehicle and *n* = 4 for ezetimibe). *Open bars*, vehicle; *gray bars*, ezetimibe (50 μg). The ^3^H-DPM abundance in each portion was shown as % as given ^3^H-DPM as 100% (*left*, *black bar*). In the vehicle treatment, almost all the tracer infused was recovered; thus, we did not measure the tracer in the cecum and data of the cecum is absent for the vehicle. In the ezetimibe treatment, the given tracer count was not sufficiently recovered. We then measured the cecum and detected approximately 20% of the given tracer in them. *B*, *Lower*; the pie charts show summaries of ^3^H distribution in the small intestine (tissue), intestinal tract (lumen), and absorbed (the sum of the serum and the liver). *C*, Dose-dependent inhibitory effect of ezetimibe on fractional cholesterol transit into the serum and the liver. The reduction in tracer activity in enterocytes reached a plateau at 5 μg ezetimibe, whereas reductions in the liver and serum were greater than that in enterocytes with 5 μg and 50 μg ezetimibe. Changes in the distribution are shown with vehicle treatment as 100%. The significance of individual differences was evaluated by using Dunnett’s test. * *p* < 0.05; ** *p* < 0.01 vs. vehicle. Bars indicate mean ± SEM (*n* = 6).

^3^H-cholesterol distribution analysis (Fig 4A) showed that the small intestine provides the large and transient reservoir, retaining 76% of tracer given orally (Fig 4B). On the other hand, the absorbed (estimated transit to the body shown in *green* in Fig 4B, *lower*) was only less than 2%, indicating that lumen-to-BBM cholesterol transit is much faster than that of enterocyte-to-circulation. Ezetimibe treatment potently reduced the absorbed fraction to less than a tenth at 50 μg dosage (Fig 4B and 4C). In contrast, the tracer abundance in the small intestine was weak, reducing only approximately by half.

With the above findings, we thought that ezetimibe potently inhibits enterocyte-to-circulation transit, but lumen-to-BBM weakly. Indeed, the efficacy of ezetimibe reached a plateau at 5 μg dosage in the isolated enterocytes (Fig 4C), while the transit to the body (the sum of the liver and the serum) was inhibited further by increasing the dosage of ezetimibe from 5 μg to 50 μg per mouse.

### Medium-to-cell cholesterol transit in HepG2 cells relies only slightly on NPC1L1

**Fig 5:**
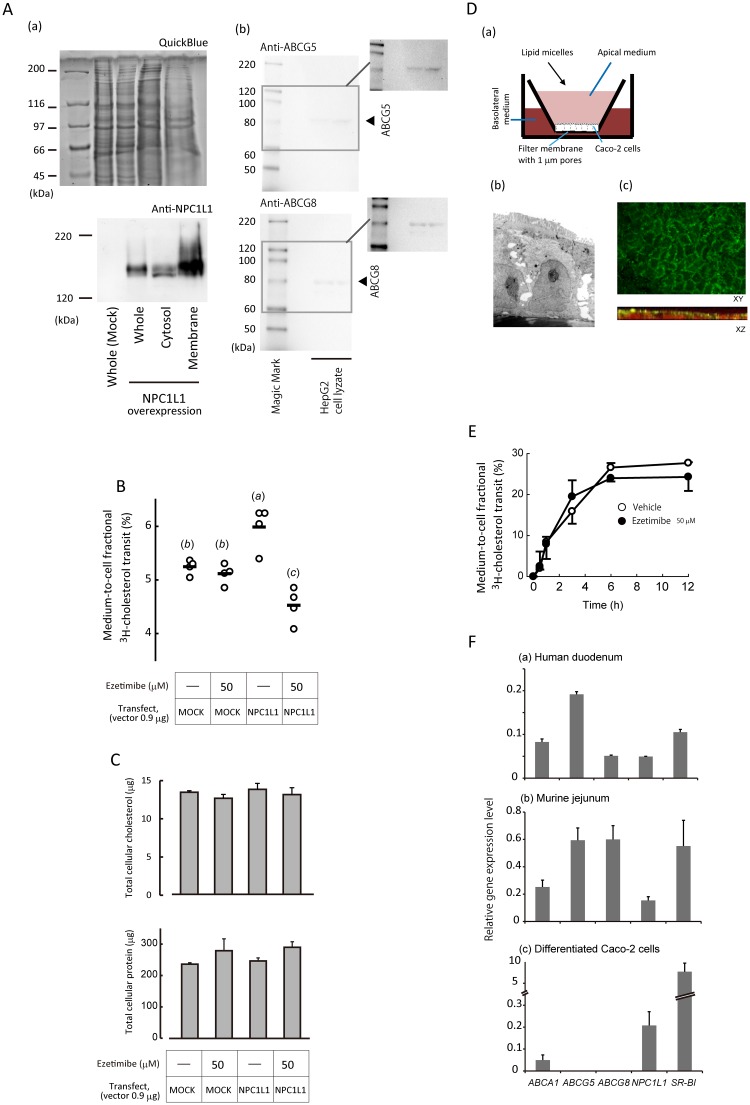
Cholesterol enters HepG2 and differentiated Caco-2 cells by NPC1L1-independent manner mainly. *A*, (a) Transient overexpression of NPC1L1 in the plasma membranes examined by Western blotting analysis. *Upper*, QuickBlue staining; *lower*, immunostaining for NPC1L1. (b) ABCG5 (*upper*) and ABCG8 (*lower*) protein expressions in HepG2 cells. *Insets*, these two images were adjusted to increase visibility of the bands. *B*, Medium-to-cell cholesterol transit in HepG2 cells. Transfection and ezetimibe treatment were performed as indicated as shown in the bottom. Medium-to-cell ^3^H-cholesterol transit efficiency (%) was estimated as described in the “Materials and Methods”. Each plot shows an individual assay result. Bars indicate means. Alphabetical differences among the groups (in parentheses) indicate significant difference between the groups (*p* < 0.05) using Tukey's Honestly Significant Difference test. *C*, Cholesterol (*upper*) and protein (*lower*) abundance in HepG2 cells. No significant difference was observed among the groups using Tukey's Honestly Significant Difference test. Bars show mean ± SEM (*n* = 4). *D*, (a) An illustration of Caco-2 cell culture system used in this study. Caco-2 cells were grown on filter membranes to allow the development to an enterocyte-like phenotype (see text for the detailed methods). The supernatants in culture inserts and wells were designated as apical and basolateral media, respectively. Lipid micelles were added to the apical medium. (b) Absorptive epithelial cell morphology of differentiated Caco-2 cell monolayers with a cylinder-like cell shape, the development of microvilli, and the distal localization of nuclei demonstrated by an electron microscopic analysis. (c) NPC1L1 was localized to the apical membrane in differentiated Caco-2 cells. Confocal microscopic analysis showed that NPC1L1 colored in *green* was localized to the plasma membrane (the upper image), especially in the brush border area (the lower image). 7-amino-actinomycin D was used for counterstaining of nuclei (*red*). *E*, Ezetimibe had little effect on medium-to-cell ^3^H-cholesterol transit in differentiated Caco-2 cells. *Open circles*, vehicle (1% ethanol); *closed circles*, 50 μmol/l ezetimibe. Data were shown as mean ± SEM of triplicate assays. *F*, In contrast to human duodenum (a) and murine jejunum (b), the gene expressions of *ABCG5* and *ABCG8* were absent in differentiated Caco-2 cells (c). Gene expression levels of five major membrane sterol transporters were analyzed by quantitative RT-PCR. An RNA sample of human duodenum was assayed in triplicate. Murine jejunal RNA samples were obtained from three C57BL/6J mice and assayed in duplicate. Three separate total RNA samples were obtained from independent wells of differentiated Caco-2 cells and assayed in duplicate. Data were shown as mean ± SEM of analytical triplicate (a) or biological triplicate (b, c) assays.

With the above findings, we wondered whether lumen-to-BBM cholesterol transit, or uptake, is the process that ezetimibe inhibits mainly. The data presented in Fig 2 suggest a possibility that the DPM reduction in the BBM of the small intestine was resulted from counteracting increased efflux. HepG2 cells lacked detectable NPC1L1 protein expression (Fig 5A). ^3^H-cholesterol was taken up by the cells without NPC1L1, indicating that the transit occurred independent of NPC1L1, probably by a diffusion manner. The overexpression of NPC1L1 increased the medium-to-cell ^3^H-cholesterol transit by 15%, showing a limited contribution of NPC1L1 in the transit. Treatment of ezetimibe (50 μmol/l, which is equivalent to 20.4 mg/kg) not only abolished the increase, but also provided further 13% reduction in the transit than mock-transfected vehicle controls. Given that medium-to-cell ^3^H-cholesterol transit by diffusion was constant, there might be a possible accelerating mechanism for cholesterol efflux in the presence of NPC1L1 together with the inhibition by ezetimibe. There was no significant difference in cellular cholesterol abundance (Fig 5C, *top*) or cytotoxicity of the drug determined by the protein abundance (Fig 5C, *lower*).

### Ezetimibe unaffected medium-to-cell ^3^H-cholesterol transit in differentiated Caco-2 cells lacking *ABCG5/G8* expressions

Differentiated Caco-2 cells expressed NPC1L1 in the apical side (Fig 5D). When ^3^H-cholesterol mixed with lipid micelles was given apically to the cells, a sufficient dosage of ezetimibe (50 μmol/l) did not inhibit the fractional medium-to-cell net ^3^H-cholesterol transit (Fig 5E). A heterodimer composed of ABCG5 and ABCG8 (ABCG5/G8) is the major cholesterol efflux transporter and should be involved in the efflux at least in a part. Quantitative RT-PCR analyses showed that differentiated Caco-2 cells lacked the gene expressions of *ABCG5* and *ABCG8* (Fig 5F). On the other hand, HepG2 cells expressed the transporter proteins [Fig 5A(b)]. The presence and absence of ABCG5/G8 might result in the difference of efficacy of ezetimibe between the two cell lines. We failed to make Caco-2 cells overexpress ABCG5/G8 proteins by conventional gene transfer techniques (data not shown) and thus could not examine whether ABCG5/G8 expressions elicit ezetimibe-mediated reduction for medium-to-cell ^3^H-cholesterol transit in Caco-2 cells. Therefore, we employed ABCG5/G8 DKO mice for further analyses as below.

### Increased cholesterol efflux is a BBM- and partly ABCG5/G8-mediated event

Lumen-to-BBM cholesterol transit was less sensitive to ezetimibe treatment, but the drug can greatly reduce the net absorption. Luminal perfusion assay showed that ezetimibe increased BBM-to-lumen cholesterol efflux. These observations suggest that the increase is counterbalancing the cholesterol fluxes at the BBM.

**Fig 6:**
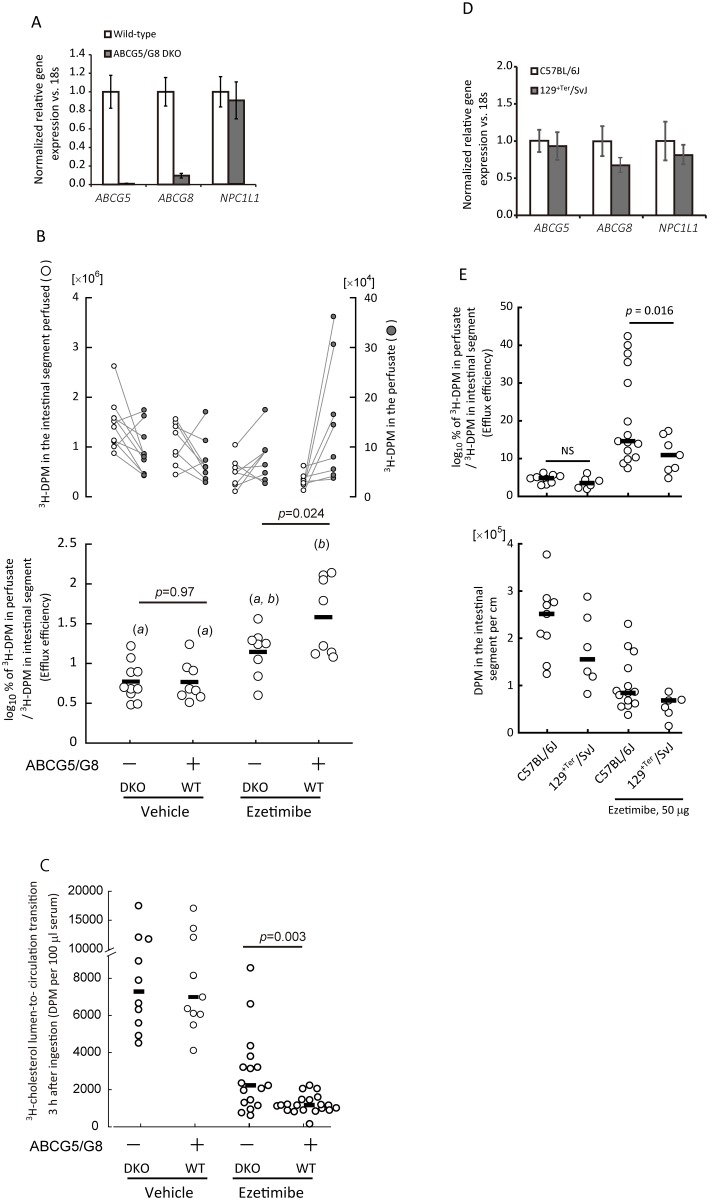
Cholesterol efflux in ABCG5 and ABCG8 double knockout (DKO) mice. *A*, Quantitative RT-PCR showed that *NPC1L1* gene expression level did not differ apparently with ABCG5/G8 deletions in mice. Gene expressions were normalized against *18s* expression levels. Then the relative expression levels were compared between the two genotypes, WT (n 10) and ABCG5/G8 DKO (n 6) with the levels in the WT mice as the references. Data are shown as mean ± SEM. *B*, Ezetimibe (50 μg)-induced increase in cholesterol efflux was partially abolished in ABCG5/G8 DKO mice. *Upper*, Plots for ^3^H-decay per minute (DPM) counts in the perfusate (*right*, *gray circles*) and the perfused intestinal segment (*left*, *open circles*). *Lower*; ^3^H-DPM in the perfusate was divided by that in the intestinal segment perfused to obtain a ratio (%), which was then converted to a common logarithm. Bars indicate medians. The difference between wild-type (WT) and DKO mice was compared using the Student’s *t*-test. Each plot shows an individual assay result. Alphabetical differences (in parentheses) indicate significant difference between the groups (*p* < 0.05) using Tukey's Honestly Significant Difference test. *C*, ABCG5/G8 DKO partially abolished the inhibitory effect of ezetimibe on the intestinal cholesterol transit. Bars indicate medians. Each plot shows an individual assay result. The difference between the WT and the DKO mice was compared using the Mann—Whitney *U*-test. *D*, Comparison of mRNA abundance by quantitative RT-PCR between the jejunal samples of C57BL/6J and 129^*+Ter*^/SvJ. Data are shown as mean ± SEM (n 5). *E*, Characteristic comparisons of C57BL/6J and 129^*+Ter*^/SvJ mice in luminal perfusion assay. *Upper*, efflux efficiency; *lower*, intestinal ^3^H-DPM abundance. The difference between the two strains was compared using the Mann—Whitney *U*-test. Bars indicate medians. Each plot shows an individual assay result.

The data obtained so far suggest that BBM-to-lumen cholesterol efflux plays a role in net cholesterol absorption and ABCG5/G8 can be involved in it. To examine whether ABCG5/G8 has a secondary role in cholesterol absorption inhibition by ezetimibe at the BBM, we performed luminal perfusion assays in ABCG5/G8 DKO and the WT mice. The lack of ABCG5/G8 did not alter *NPC1L1* gene expression level significantly, excluding adaptive or compensatory modulation of *NPC1L1* expression (Fig 6A). Luminal perfusion assays showed that the presence of ABCG5/G8 enhanced the ezetimibe-stimulated increase in efflux from 4.0-fold to 10-fold, without a significant difference in the abundance of intestinal ^3^H-cholesterol labeling between the genotypes [3.2 × 10^5^ ± 0.05 × 10^5^ for the ezetimibe-treated WT mice vs. 4.7 × 10^5^ ± 1.0 × 10^5^ for the ezetimibe-treated DKO mice, in DPM (mean ± SEM); *p* = 0.42 by the Student-*t* test, Fig 6B, *upper*]. In the absence of ezetimibe, cholesterol efflux did not differ between the DKO and the WT mice (*p* = 0.97; Fig 6B, *lower*).

### Larger variability of efflux efficiency in B6; 129S6-*Abcg5*/*Abcg8*^*tm1Hobb*^/J mice

B6; 129S6-*Abcg5*/*Abcg8*^*tm1Hobb*^/J mice had greater variability than C57BL/6J mice in the efficiency of cholesterol efflux and fractional cholesterol transit (Figs 2C and 6B). These mice have a genetically heterogenic background of 129 and B6 lines; thus, we thought that the heterogeneous genetic background among the transgenic mice might explain the greater assay variability. By using C57BL/6J and 129^*+Ter*^/SvJ mice as representative B6 and 129 lines, respectively, we observed that 129^+Ter^/SvJ mice had similar mRNA abundances for *ABCG5*, *ABCG8*, and *NPC1L1* to C57BL/6J mice (Fig 6D). Although there was no significant difference in the basal cholesterol efflux between the two strains, 129^*+Ter*^/SvJ mice had a lower response to ezetimibe than C57BL/6J mice (Fig 6E, *upper*). 129^+*Ter*^/SvJ mice had relatively lower labeling, but there was also no statistical difference in intestinal ^3^H-cholesterol labeling between 129^*+Ter*^/SvJ and C57BL/6J mice (Fig 6E, *lower*). These observations might partly explain the greater data variability, but the major cause is still unknown.

### Efficiency of fractional cholesterol absorption and efflux are inversely correlated

**Fig 7:**
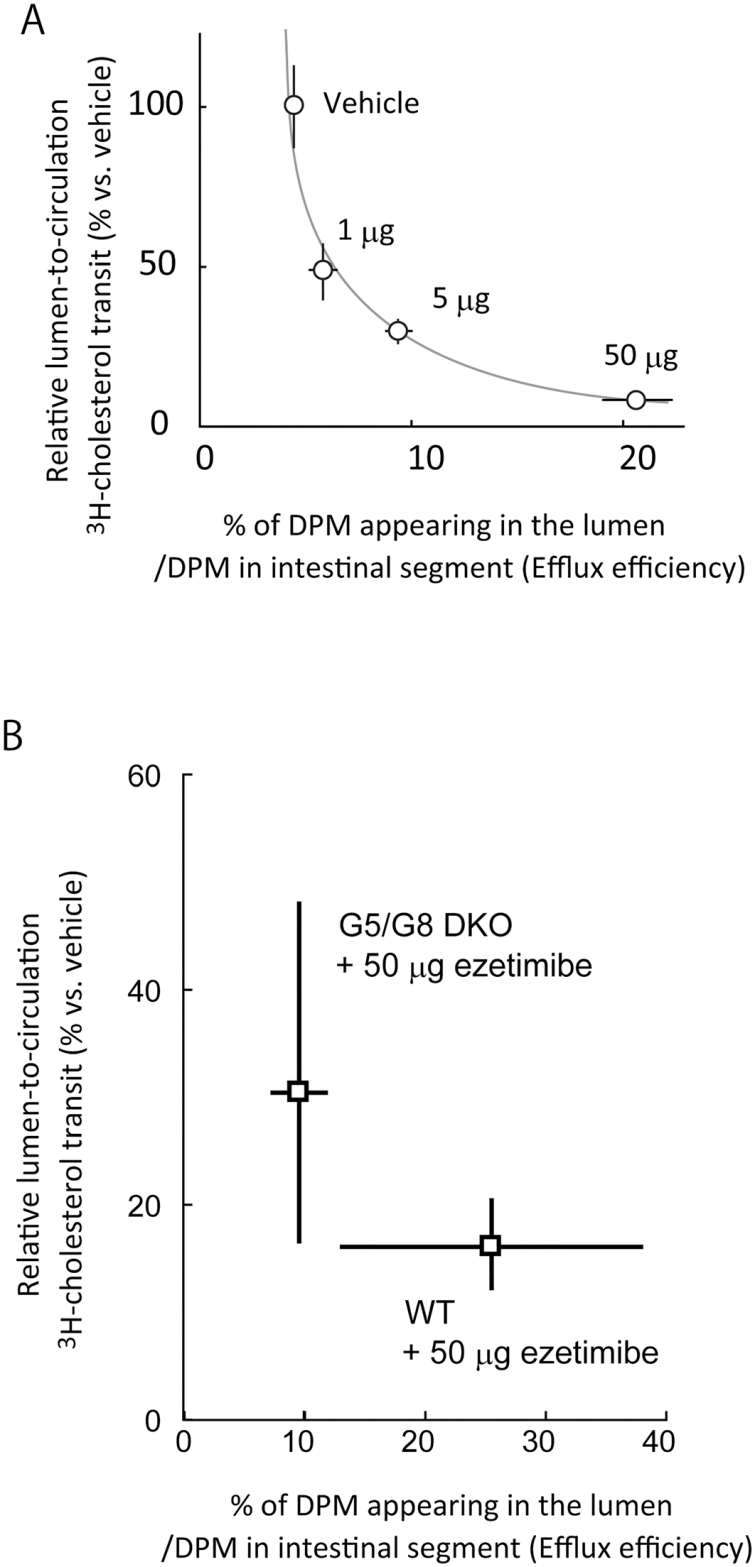
An inverse correlation between BBM-to-lumen cholesterol efflux and fractional lumen-to-circulation transit. *A*, Comparison of fractional cholesterol transit (Fig 4C) and cholesterol efflux efficiency (Fig 2C) in ezetimibe-treated C57BL/6J mice. *B*, Comparison of fractional cholesterol transit and cholesterol efflux efficiency between WT and ABCG5/G8 DKO mice presented in Fig 5A, *lower*, and 5B. *A* and *B*, The mean relative efflux efficiency (%) and the median relative absorption (%) versus vehicle are plotted. Bars for the X-axis and Y-axis indicate the SEM and 50% coefficient interval, respectively.

Ezetimibe inhibited enterocyte-to-circulation transit (Fig 4C). In so doing, the drug increased BBM-to-lumen cholesterol efflux of dietary origin (Fig 2). When we compared the efflux efficiency (Fig 2C) and fractional cholesterol transit to the circulation (Fig 4C), an inverse relationship between the two parameters was observed (Fig 7A). The relationship indicates that BBM-to-lumen cholesterol flux is a determinant of cholesterol absorption in addition to lumen-to-BBM and enterocyte-to-circulation transits. Furthermore, overexpression of ABCG5/G8 reduced fractional cholesterol absorption in the small intestine [20]. Luminal perfusion assays in mice showed that ABCG5/G8 mediates a rapid efflux (Fig 6C), increasing efflux efficiency by approximately 2.5-fold (Fig 6C), consistent with the functional role in the efflux and the counteracting effect on cholesterol absorption. These data are also summarized in Fig 7B.

### Plant sterol absorption and the efflux are also inversely correlated

The inverse relationship proposed in Fig 7 can be ezetimibe-specific phenomena. To show that such complement in flux is not restricted to, and occurs without ezetimibe, we employed plant sterols, which have a similar physicochemical nature to cholesterol but poorly absorbable from the small intestine. We used radiolabelled tracers of sitosterol and sitostanol, a weak [21, 22] and blunt substrate for NPC1L1, respectively. ABCG5/G8 pumps out cholesterol and sitosterol similarly, suggesting that the transporter is sterol-nonselective [23]. Thus, plant sterol handlings should be similar to cholesterol handling in the small intestine, but the major difference is how much the sterol is forwarded to be absorbed with relatively cholesterol specific machineries, such as NPC1L1 and acyl-CoA acyltransferase 2 of the enterocytes.

**Fig 8:**
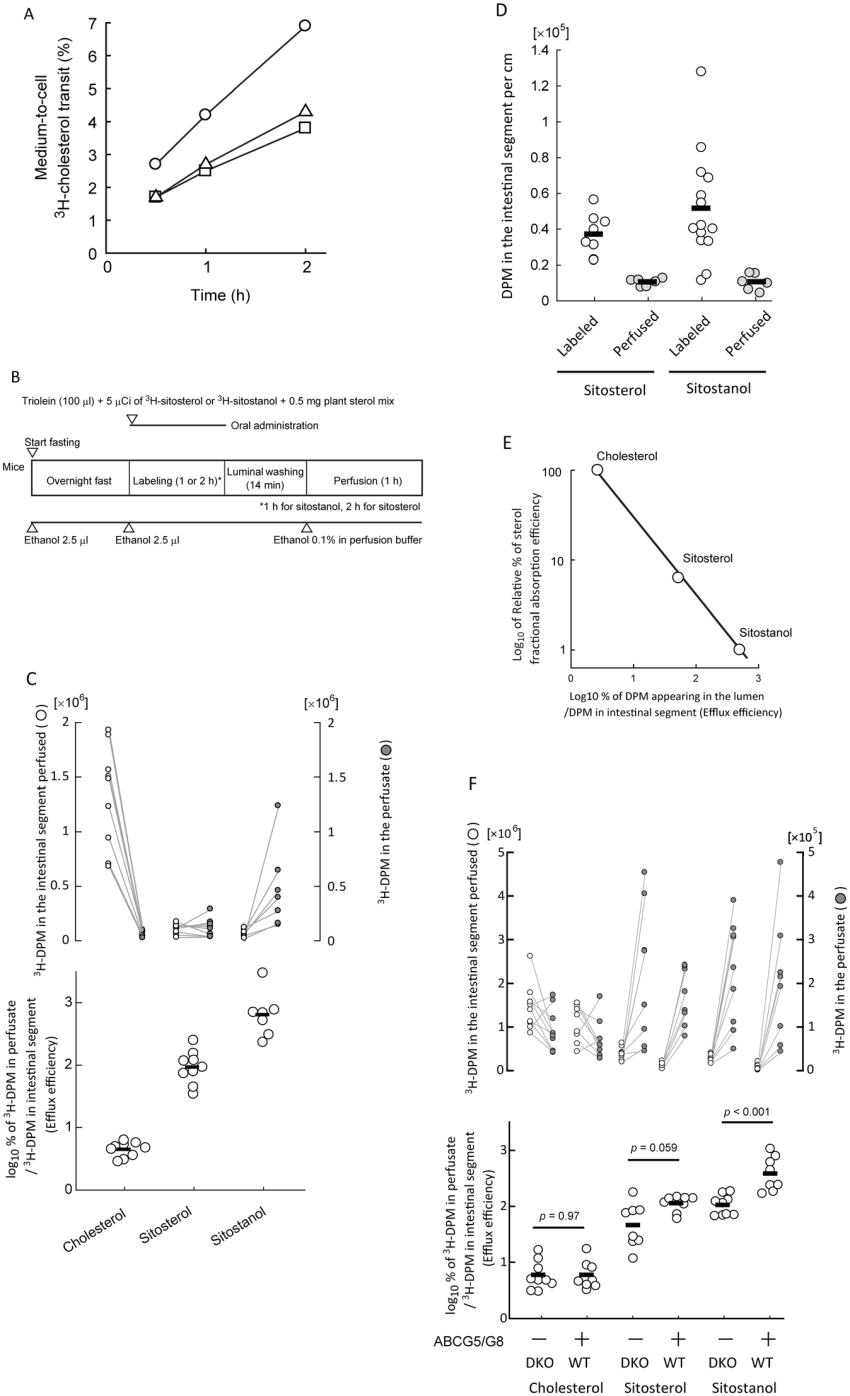
Poorly absorbable sitosterol and sitostanol show greater efflux efficiencies than cholesterol. *A*, Time-course uptake of ^3^H-cholesterol (*circles*), ^3^H-sitosterol (*squares*), and ^3^H-sitostanol (*triangles*) by differentiated Caco-2 cells. Plots are shown as mean of duplicate assays. *B*, An illustrated protocol for the luminal perfusion assay with sitosterol or sitostanol tracers. Assay time course is indicated from left to right. Open triangles show time points when reagents were given to mice. *C*, *Upper*; Plots for ^3^H-decay per minute (DPM) counts in the perfusate (*right*, *gray circles*) and perfused intestinal segment (*left*, *open circles*). *Lower*; Comparison of efflux efficiency of three tracers: cholesterol, sitosterol, and sitostanol. *D*, Change in ^3^H abundance per cm of intestinal segment during luminal perfusion assay with ^3^H-sitosterol or ^3^H-sitostanol. Bars indicate medians. Each plot shows an individual assay result. *E*, Comparison of sterol absorption efficiency (cholesterol absorption efficiency as 100%) with efflux efficiency for cholesterol, sitosterol, and sitostanol. Sterol absorption efficiencies were obtained from Igel *et al*. (Ref. 10), in which absorption efficiencies for sitosterol and sitostanol were estimated as 7% and 1%, respectively. Efflux efficiencies were obtained from *C*. *F*, Efflux efficiency of three sterol tracers in ABCG5/G8 double knockout (DKO) mice. *Upper*; Plots for ^3^H-DPM counts in the perfusate (*right*, *gray circles*) and perfused intestinal segment (*left*, *open circles*). *Lower*; Effect of ABCG5/G8 DKO on sterol efflux efficiency. *P* values were obtained using the Student’s *t*-test (sitostanol and cholesterol) or the Mann—Whitney *U* test (sitosterol) for comparison of efflux efficiency. Each plot shows an individual assay result.

Medium-to-cell ^3^H-choelsterol transit assay showed that the three sterols tested were taken up by the BBM comparable to cholesterol in differentiated Caco-2 cells (Fig 8A). Luminal perfusion assays with ^3^H-sitosterol and ^3^H-sitostanol in C57BL/6J mice (Fig 8B) showed that the median efflux efficiencies were 52-fold and 497-fold, respectively, greater than that with ^3^H-cholesterol (Fig 8C). We confirmed that the intestinal segments perfused were labelled with plant sterol tracers and most of these labelings were disappeared during the perfusion assay (Fig 8D), suggesting that the losses were due to the large effluxes to the lumen.

In C57BL/6J mice, fractional sitosterol and sitostanol lumen-to-BBM transit efficiencies were reported to be ~7% and ~1%, respectively, compared with that of cholesterol [10]. Comparison of the transit efficiencies and the efflux efficiencies showed inverse relationship between the two parameters (Fig 8E). Luminal perfusion assays showed that the effluxes of sitosterol and sitostanol were increased by 41% and 29%, respectively, in the presence of ABCG5/G8 (Fig 8F).

## Discussion

In the present study, we directly detected ezetimibe-stimulated increase in TICE, or circulation-to-lumen cholesterol efflux, for the first time. Luminal perfusion assays with orally-infused ^3^H-cholesterol labeling indicated that the ezetimibe-stimulated TICE increase was a result of the promotion of BBM-to-lumen cholesterol efflux. Further analyses revealed an inverse relationship between intestinal cholesterol efflux and lumen-to-circulation fractional cholesterol transit, showing that the efflux is a determinant for cholesterol absorption efficiency.

### Ezetimibe increases BBM-to-luminal cholesterol efflux

We asked how inhibition of the influx transporter NPC1L1 by ezetimibe can increase cholesterol efflux (Fig 1). As a hydrophobic substance, cholesterol has high affinity to the BBM. Indeed, ezetimibe did not inhibit cholesterol entering the BBM of differentiated Caco-2 cells (Fig 5E). These suggest that lumen-to-BBM cholesterol transit is not sensitive to ezetimibe. Chang and Chang [24] suggested two possible mechanisms for how NPC1L1 binds to luminal cholesterol: One is that NPC1L1 directly binds to cholesterol present in the mixed micelles in the lumen; the other is that cholesterol binds to NPC1L1 after entering the BBM. The results of the present study are consistent with the latter possibility. In fact, ezetimibe appears to reduce the absorption by inhibiting the internalization from the BBM to the cell interior [21, 25, 26]. This inhibition, in turn, would direct excess cholesterol in the BBM to be effluxed back to the lumen not to disturb the membrane integrity.

### ABCG5/G8 promotes rapid BBM-to-lumen cholesterol efflux

Studies showed that genetic inactivation of ABCG5/G8 did not affect cholesterol absorption [27, 28], basal TICE [2], or FNS excretion [6]. Similarly, we observed unaltered BBM-to-lumen cholesterol efflux with the deletion of ABCG5/G8 in the absence of ezetimibe (Fig 8F). These suggest that basal efflux does not depend on ABCG5/G8. On the contrary, ABCG5/G8 promotes ezetimibe-stimulated BBM-to-lumen cholesterol efflux (Fig 6) or poorly absorbable non-cholesterol sterols were applied (Fig 8). Consistent with the condition dependency, Jakulj et al. showed that the genetic deletion of *ABCG8* almost abolished increase in FNS excretion especially in the ezetimibe-treated mice [6].

Our findings suggest that inhibition of NPC1L1 results in accumulation of cholesterol in the BBM momentarily, and the accumulation increases the chance to interact sterols with ABCG5/G8 to be effluxed back to the lumen at the cholesterol-rich microdomains of the BBM [29], where the two transporters are localized. Also, this elimination mechanism within the BBM may underlie the ABCG5/G8-involved protection against a variety of hydrophobic and potentially toxic non-cholesterol sterols contained in meals abundantly [30].

NPC1L1 discriminates, but not completely, cholesterol from structurally-related non-cholesterol sterols by the binding affinity [31]. Without functional ABCG5/G8, non-cholesterol sterols can still enter and accumulate in the body [28, 32]. Ezetimibe reduces plasma plant sterol levels in mice lacking *ABCG5/G8* and patients with homozygous phytosterolemia and can improve phytosterolemia-related adverse conditions, but the levels remain substantially above the normal range [27, 32]; at an average of ~50% of pretreatment levels in the patients even with years of the treatment with the drug [33, 34].

The above clinical and experimental outcomes suggest a possibility that (*i*) ABCG5/G8, a non-selective sterol drain to the lumen, lowers the threshold in eliminating sterols from the BBM; (*ii*) ABCG5/G8 maintains sterol selectivity in the BBM at a reliable level together with an incomplete sterol selector, NPC1L1; (*iii*) cholesterol concomitantly spills out from the BBM by diffusion or ABCG5/G8 into the lumen, resulting in reduced (or regulated) cholesterol absorption efficiency. Rapid plant sterol efflux occurred even without ABCG5/G8, but it diminished (Fig 8F). Although the absorption efficiency is much smaller than cholesterol, the reduction in efflux should be associated with intolerable plant sterol absorption and accumulation in the body.

### Brush border membrane as a platform for bidirectional cholesterol flux

Inhibition of the cholesterol influx transporter NPC1L1 increased BBM-to-lumen efflux; conversely, deletion of ABCG5/G8, an efflux heterodimer transporter, increased fractional cholesterol absorption. These show that the inhibition of efflux promotes the opposite influx, and *vice versa* (Fig 6).

**Fig 9:**
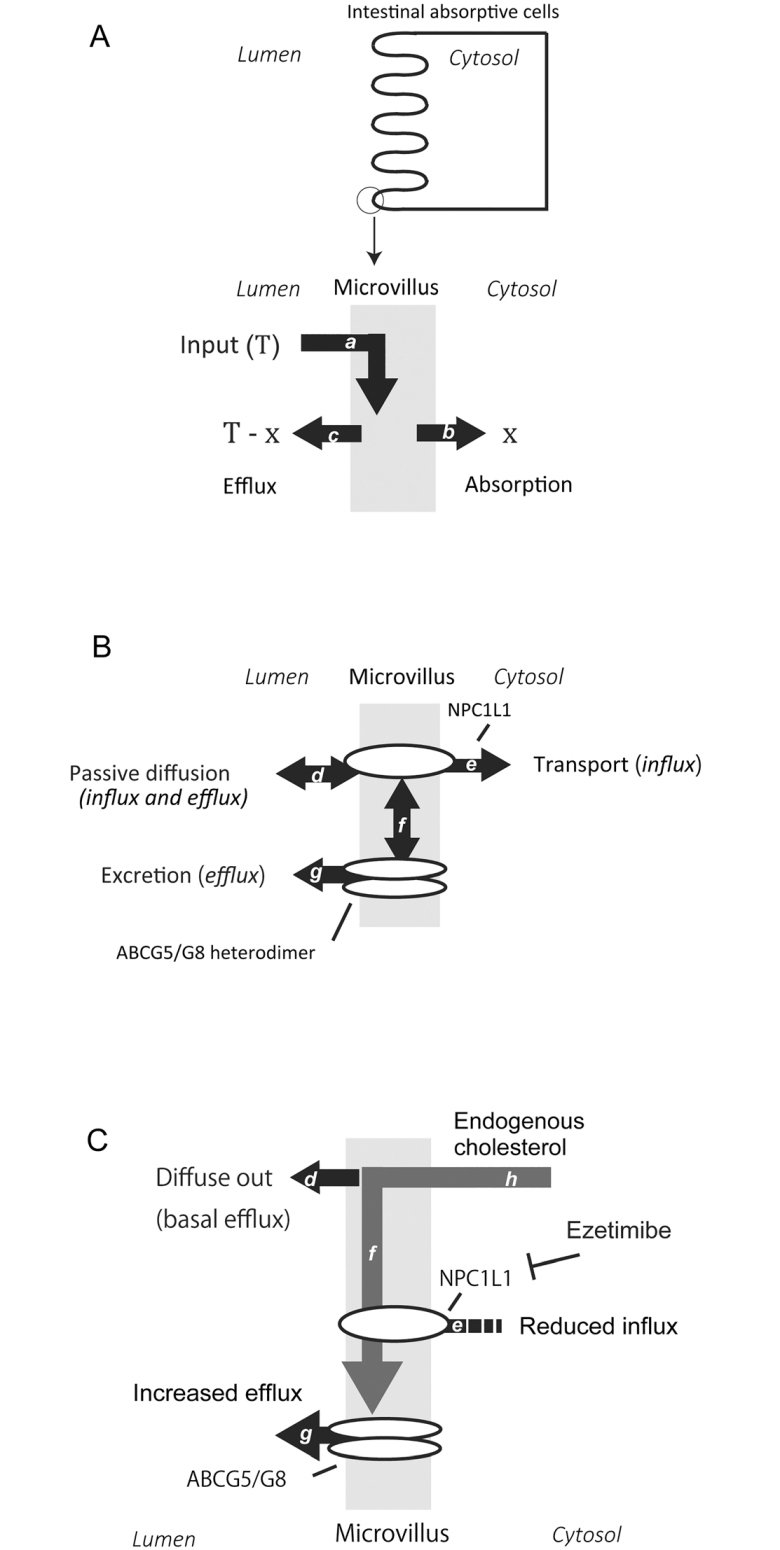
Brush border membrane as a major platform for cholesterol bidirectional flux. *A*, Balance out of cholesterol translocation in the brush border membrane (BBM). The BBM is focused (the circle in the upper panel); the gray square indicates lipid bilayer, or microvillus. *T*, total input cholesterol incorporated into the BBM from the apical side. Cholesterol (*T*) diffuses into the BBM via the pathway (*a*). *x*, the amount of cholesterol transferred to endosomes for further processing via the pathway (*b*) and considered to be transferred eventually to the circulation, which of the following processes were not identified in this presentation. *T—x*, estimated amount of cholesterol effluxed into the lumen via the pathway (*c*). Thus, ‘*x*’ and ‘*T—x*’ should be inversely correlated. *B*, An illustration of the hypothesized bidirectional intestinal cholesterol flux system. Cholesterol diffuses into the BBM (pathway *d*; Fig 6); NPC1L1 mediates cholesterol movement from the BBM to the endosomal processing [*e*; Chang T-Y and Chang C. (2008, Ref. 24)]; Cholesterol that diffuses into the BBM can be caught by ABCG5/G8 in cholesterol-rich microdomains (*f*). ABCG5/G8 promotes elimination of sterols from the BBM when required or the sterols become in excess (*g*; Fig 6). Collectively, the BBM provides a buffering space for the bidirectional flux of cholesterol. *C*, A proposed route for TICE. Endogenous cholesterol circulates into the BBM (pathway, *h*). Ezetimibe prevents internalization of the cholesterol from the BBM (*e*) (Ref.24). The cholesterol in the BBM diffusively exits to the lumen or is pumped out by ABCG5/G8 (*d* and *g*).

Given that ezetimibe does not prevent cholesterol from entering the membrane (***T***), cholesterol transit to cell interior (***x***) and cholesterol efflux (***T—x***) from the membrane can be correlated inversely to counterbalance the flux (Fig 9A). Consistently, we confirmed an inverse relationship between cholesterol efflux and fractional cholesterol transit in the presence of gradual dosages of ezetimibe (Fig 7A) and in the examination with three sterol tracers that have different absorption efficiency (Fig 8E). Therefore, such relationship again indicates that cholesterol efflux is a determinant for sterol absorption. We adapted the framework to the BBM having NPC1L1 and ABCG5/G8 (Fig 9B).

In luminal perfusion assay, ezetimibe-stimulated increase in efflux was disappeared rapidly (Fig 2E), probably by the depletion of ^3^H-cholesterol tracer in the lumen that supplies the BBM with the tracer continuously (pathway ***d*** in Fig 9B). This suggests that the sterol balance and the composition in the BBM are maintained strictly and adjusted in minutes. In fact, plant sterol tracers incorporated into the BBM were almost effluxed back to the lumen in luminal perfusion assay (Fig 7D). Most of these removal occurred in the beginning of the assay period (data not shown).

### Intestinal cholesterol flux modulation by other compounds

Ezetimibe inhibits cholesterol absorption and increases BBM-to-lumen efflux. Such bilateral effects were also observed with the other compounds. Plant sterols, a dietary cholesterol absorption inhibitor, stimulated FNS excretion more than 3-fold and the estimated increase in intestinal cholesterol excretion was approximately 5-fold [35]. Liver-X-receptor agonists, which establish efflux-prone conditions in the small intestine by modulating the gene expression of cholesterol transporters, not only reduced intestinal cholesterol absorption [36, 37] but also increased macrophage-to-feces RCT [38]. These consistent observations indicate that the bidirectional flux system via the BBM may not be unique to the conditions that use ezetimibe as a treatment.

### Increased reverse cholesterol transport by ezetimibe

We have shown that TICE was enhanced by ezetimibe probably as a consequence of increased BBM-to-lumen cholesterol efflux in mice. The intestinal RCT probably takes a route of circulation-to-feces via the BBM as the intermediate (Fig 9C). This would explain hepato-biliary-independent increase in FNS excretion in ezetimibe-treated mice [6, 28, 39].

We do not intend to exclude hepatic components in the increased FNS excretion by ezetimibe. The levels of NPC1L1 gene expression in the liver and the binding affinity of ezetimibe to NPC1L1 differ considerably among species [3, 9]. This should result in the varying contribution of intestine-mediated FNS excretion in the total cholesterol disposal and inconsistent effect of ezetimibe among species. Indeed, Uto-Kondo et al. showed a hepatic contribution in ezetimibe-stimulated RCT in hamsters [40]. Also, intestinal RCT may depend on the cholesterol handling in the circulation and lipoprotein composition. High-density lipoprotein plays a key role in transferring macrophage-derived cholesterol to the liver. In TICE, on the other hand, high-density lipoprotein is not likely a dominant or exclusive lipoprotein to supply enterocytes with cholesterol [41, 42].

### Study limitations

We suggest that ezetimibe increases TICE by stimulating BBM-to-lumen cholesterol efflux and assum that cholesterol in the BBM can be supplied from the circulating cholesterol; however, the entire route from the circulation to the lumen has not been determined. For example, it is still incompletely understood how cholesterol is transferred to the apical membrane. Also, there may be other pathways that ezetimibe affects among the process.

The radiolabelled cholesterol should behave similarly to endogenous cholesterol; thus, we thought that net cholesterol flux could be estimated by the tracer counting. Our data provide the flux efficiencies in a comparative manner, but not a quantitative manner. We did not determine cholesterol quantity in each sample, because quantitative approach can be problematic in our assay settings. The BBM itself has abundant in cholesterol and part of it can slough into the lumen, for examples. To quantitate the impact of TICE on eventual cholesterol disposal, indirect calculation of input (cholesterol in diet, the bile, and intestinal cell debris) and output (FNS excretion) should be effective.

It is still ambiguous how NPC1L1 handles luminal cholesterol. The present study did not intend to define the mechanism; however, our physiological data suggested that luminal cholesterol binds to NPC1L1 after entering the BBM diffusively and that ABCG5/G8 plays a role in the efflux especially when the function of NPC1L1 is impaired. Further careful approaches should be necessary to determine the mechanism.

## Conclusions

The results of the present study suggest that BBM-to-lumen cholesterol efflux plays a role in TICE and regulating net cholesterol absorption. The BBM provides a buffering space for those cholesterol fluxes in the small intestine. The observations integrate the phenomena of intestinal cholesterol efflux, cholesterol absorption, TICE, and presumably eventual intestine-mediated increase in FNS excretion, into a single system with the BBM as a major platform (Fig 9B). The integration would provide a framework to make it clear the elusive cholesterol flux in the small intestine, and explore ways modulating it to dispose of endogenous cholesterol efficiently for therapeutic purposes.

## Supporting Information

S1 Table Primer pairs for human genes used in this study. (PDF)

S2 Table Primer pairs for mouse genes used in this study. (PDF)
