# Supplementary material for: Ezetimibe Promotes Brush Border Membrane-to-Lumen Cholesterol Efflux in the Small Intestine
Source: PLoS One. 2016 Mar 29;11(3):e0152207. doi: 10.1371/journal.pone.0152207 (PMC4811413; doi:10.1371/journal.pone.0152207)
Supplement: S1 Table — (PDF) [file pone.0152207.s001.pdf]

S1 Table. Primer pairs for human genes used in this study.

HGNC symbol	Accession No.	Name	Forward primer (5'-3')	Reverse primer (5'-3')	Refs.
ABCA1	NM_005502.2	ATP-binding cassette A1	ATGTCCAGTCCAGTAATGGTTCTGT	CGAGATATGGTCCGGATTGC	(1)
ABCG5	NM_022436.2	ATP-binding cassette G5	GCATGCTGAACGCTGTGAA	TGGTAGAGGCCGTCCTGACT	(1)
ABCG8	XM_055525.1	ATP-binding cassette G8	AGCCGCCCTCTTGTTCATG	GAGTAACATTTGGAGATGACATCCA	(1)
B2M	NM_004048	β 2-microglobulin	ACTGAATTCACCCCCACTGA	CCTCCATGATGCTGCTTACA	(2)
NPC1L1	NM_013389.2	NPC1 (Niemann-Pick disease, type C1, gene)-like 1	ACATCAGCGTGGGACTGG	AGTCAAGCAGGTACGAGTCCTT	*1
SCARB1	NM_005505.4	Scavenger Receptor B1	CATCAAGCAGCAGGTCCTTA	CGGAGAGATAGAAGGGGATAGG	*1

*1, The primer sets were designed with using Assay Design Center at http://www.roche-applied-science.com/.

1. Field, F. J., E. Born, and S. N. Mathur. 2004. LXR/RXR ligand activation enhances basolateral efflux of β-sitosterol in CaCo-2 cells. *Journal of Lipid Research* **45**: 905-913.

2. Galiveti, C. R., T. S. Rozhdestvensky, J. Brosius, H. Lehrach, and Z. Konthur. 2010. Application of housekeeping npcRNAs for quantitative expression analysis of human transcriptome by real-time PCR. *RNA* **16**: 450-461.
