# Supplementary material for: Ezetimibe Promotes Brush Border Membrane-to-Lumen Cholesterol Efflux in the Small Intestine
Source: PLoS One. 2016 Mar 29;11(3):e0152207. doi: 10.1371/journal.pone.0152207 (PMC4811413; doi:10.1371/journal.pone.0152207)
Supplement: S2 Table — (PDF) [file pone.0152207.s002.pdf]

S2 Table. Primer pairs for mouse genes used in this study.

HGNC symbol	Accession No.	Name	Forward primer (5'-3')	Reverse primer (5'-3')
18S	NR_003278.3	18S ribosomal RNA	CGGCTACCACATCCAAGGAA	GCTGGAATTACCGCGGCT
ABCA1	NM_013454.3	ATP-binding cassette A1	GCCCATCATCTGCCAACT	TCCTGGGAGCCCTTTTTACT
ABCG5	NM_031884.1	ATP-binding cassette G5	TGAGCTGCCCTTTCTGAGTC	GGAGCTCAGAGACCCTCTGTT
ABCG8	NM_026180.2	ATP-binding cassette G8	AACCCTGCGGACTTCTACG	CTGCAAGAGACTGTGCCTTCT
NPC1L1	NM_207242.2	NPC1 (Niemann-Pick disease, type C1, gene)-like 1	TTCTTGCCCACAGTACCCCTT	CTCGTGGCCCCTTATCAGC
SCARB1	NM_016741.1	Scavenger Receptor B1	GCCCATCATCTGCCAACT	TCCTGGGAGCCCTTTTTACT

The primer sets were designed with using Assay Design Center at http://www.roche-applied-science.com/.
